# Translational fidelity screens in mammalian cells reveal eIF3 and eIF4G2 as regulators of start codon selectivity

**DOI:** 10.1093/nar/gkad329

**Published:** 2023-05-05

**Authors:** Richard She, Jingchuan Luo, Jonathan S Weissman

**Affiliations:** Whitehead Institute for Biomedical Research, Cambridge, MA, USA; Whitehead Institute for Biomedical Research, Cambridge, MA, USA; Whitehead Institute for Biomedical Research, Cambridge, MA, USA; Department of Biology, Massachusetts Institute of Technology, Cambridge, MA, USA; David H. Koch Institute for Integrative Cancer Research, Massachusetts Institute of Technology; Cambridge, MA 02142, USA; Howard Hughes Medical Institute, Massachusetts Institute of Technology, Cambridge, MA, USA

## Abstract

The translation initiation machinery and the ribosome orchestrate a highly dynamic scanning process to distinguish proper start codons from surrounding nucleotide sequences. Here, we performed genome-wide CRISPRi screens in human K562 cells to systematically identify modulators of the frequency of translation initiation at near-cognate start codons. We observed that depletion of any eIF3 core subunit promoted near-cognate start codon usage, though sensitivity thresholds of each subunit to sgRNA-mediated depletion varied considerably. Double sgRNA depletion experiments suggested that enhanced near-cognate usage in eIF3D depleted cells required canonical eIF4E cap-binding and was not driven by eIF2A or eIF2D-dependent leucine tRNA initiation. We further characterized the effects of eIF3D depletion and found that the N-terminus of eIF3D was strictly required for accurate start codon selection, whereas disruption of the cap-binding properties of eIF3D had no effect. Lastly, depletion of eIF3D activated TNFα signaling via NF-κB and the interferon gamma response. Similar transcriptional profiles were observed upon knockdown of eIF1A and eIF4G2, which also promoted near-cognate start codon usage, suggesting that enhanced near-cognate usage could potentially contribute to NF-κB activation. Our study thus provides new avenues to study the mechanisms and consequences of alternative start codon usage.

## Graphical Abstract

**Graphical Abstract:**
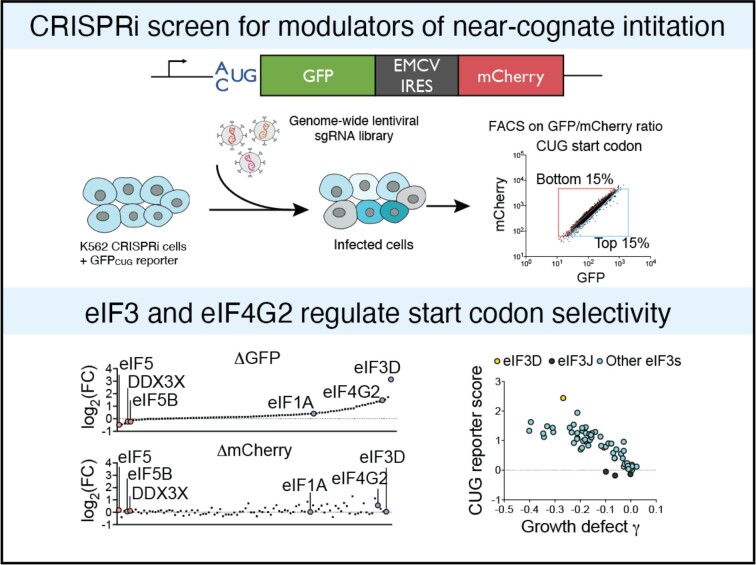
Genome-wide CRISPRi screening reveals modulators of CUG near-cognate start coding usage in mammalian cells.

## INTRODUCTION

The fidelity of transcription and translation ensures the faithful transmission of genetically encoded DNA sequence into functional protein. The accuracy of each step requires biochemical discrimination between individual nucleotides or triplet codons. Whereas DNA replication introduces errors at rates as low as 1 in 10^8^ bp after mismatch repair (1), translation is the most error prone and energetically costly step in protein production (2,3). However, because mistranslation events are only transiently encoded in protein products that can be readily degraded, quantifying the exact rates and modalities of aberrant translation in eukaryotic cells remains highly challenging.

The production of protein from mRNA is typically initiated at an AUG start codon, which ensures that translation begins in the proper reading frame. However, a small handful of endogenous proteins are exclusively initiated at non-AUG start codons, including eIF4G2, which is initiated at an evolutionarily conserved GUG start codon (4,5). Experiments to systematically document the full breadth of translation products within a cell have been enabled by ribosome profiling (6,7). In particular, high-resolution ribosome footprinting identified numerous noncanonical translation products, including upstream open reading frames (uORFs), which were initiated at both AUG and non-AUG (near-cognate) start codons. While translation initiation at near-cognate start codons is generally biochemically disfavored, CUG and GUG start codons are the most commonly found and efficiently utilized near-cognate start codons (8). Initiation at uORFs can modulate the expression of the downstream main ORF and empirically constrains the length of 5′UTRs (9). In the human genome, 5′UTRs are only 210 bp on average, whereas 3′UTRs are 1028 bp on average (10). Furthermore, the regulatory effect of uORF initiation has been well documented in the ATF4 and BiP transcripts (11,12), which act as key mediators of the integrated stress response. Recent evidence has emerged that the peptide products of uORFs can possess intrinsic functions (13,14). Many such micropeptides exerted effects on cellular growth and functional characterization revealed several with distinct cellular localizations and protein binding partners. Lastly, hundreds of uORF protein products were found to be presented by the MHC-I, suggesting that these peptides could comprise a meaningful fraction of the cell's antigen repertoire (13,15,16).

The biochemical machinery that orchestrates start codon recognition has been characterized in great detail. In eukaryotes, variants of the consensus Kozak sequence (GCCACCAUGG) are commonly found directly upstream of both canonical AUG and near-cognate start codons (17). In addition, numerous highly conserved initiation factor complexes play essential roles in directing the start of translation. In the classic model of translation, an initiator methionine tRNA (Met-tRNA_i_) is bound by eIF2 and loaded onto the 40S small ribosomal subunit. This process is guided by physical interactions with eIF1, eIF1A, eIF3 and eIF5, and these factors collectively comprise the 43S pre-initiation complex (PIC) (18,19). The 43S PIC is then recruited to the 5’-proximal region of the mRNA via interactions with several cap-associated initiation factors and begins scanning in the 5’ to 3’ direction until it reaches a start codon with a suitable local nucleotide context (20–22). Base-pairing between Met-tRNA_i_ and start codon facilitates a conformational rearrangement of the scanning complex and joining of the 60S large ribosomal subunit to form the 80S ribosome, which then begins the process of translation. These final steps in start codon selection are regulated by displacement of eIF1 from the ribosomal P-site, eIF5-induced hydrolysis of eIF2•GTP, and eIF5B-mediated joining of the 60S (23–28). Previous genetic reversion screens in *S. cerevisiae* identified eIF1, eIF1A, eIF2 and eIF5 as genes involved in rescuing expression of an auxotrophic marker driven by a near-cognate start codon (26,29–32). Similarly, biochemical assays in rabbit reticulocyte lysate showed that increased stoichiometry of eIF1, eIF1A, eIF5 and eIF5B altered the frequency of near-cognate start codon usage (8,33). Lastly, targeted mutagenesis of regions of eIF3C and eIF4G known to interact with eIF1 and eIF5 led to mutants capable of promoting near-cognate start codon usage (34–37).

We set out to explore whether near-cognate initiation in mammalian cells could be regulated or perturbed by intrinsic cell biological processes. To do so, we performed genome-wide CRISPR interference (CRISPRi) screens in human K562 cells expressing a fluorescent reporter encoded with a CUG near-cognate start codon. The strongest genes identified by our unbiased screening approach recapitulated genetic reversion screens in yeast and included roles for eIF3 and eIF4G2 in regulating near-cognate start codon usage. Follow-up experiments showed that alternative cap-binding by eIF3D/eIF4G2 was not required but that the N-terminus of eIF3D was essential for rescuing normal start codon stringency. Lastly, transcriptional profiling of cells depleted for eIF1A, eIF3D and eIF4G2 revealed activation of NF-κB targets and the interferon gamma response.

## MATERIALS AND METHODS

### Reagents

**Table:** 

Component	Manufacturer	Product #
Illumina TruSeq Stranded Total RNA kit	Illumina	20020599
NEBuilder HiFi DNA Assembly Kit	NEB	E2621X
Mirus Transfection Reagent	VWR	10767–122
Polybrene	Millipore Sigma	107689
1L spinner flask	DWK Life Sciences	356884
1x ViralBoost	Alstem	VB100
RPMI media	Thermo	22400105
FBS	VWR	97068–085, lot 043K20
Penicillin-streptomycin-glutamine	Gibco	10378016
DMEM media	Thermo	11965118
0.45 μm filter	Millipore Sigma	SLHP033RS
Puromycin	Goldbio	*P*-600–100
Mesh cap tube	Corning	352235
Macherey-Nagel NucleoSpin Blood XL kit	Thermo	NC1105387
NEBNext Ultra II Q5 Master Mix	Thermo	50–105-0634
SPRIselect magnetic beads	Thermo	NC0406407
T4 DNA ligase	NEB	M0202S
Stellar Competent Cells	Takara Bio	636766
Qubit High Sensitivity dsDNA assay	Thermo	Q32854
Direct-zol RNA miniprep	Zymo Research	ZR2052
High Sensitivity DNA Bioanalyzer Kit	Agilent	5067–4626
10% SDS	Ambion	AM9822
0.5 M EDTA	Ambion	AM9260G
SuperScript IV VILO	ThermoFisher	11756050
DyNAmo ColorFlash SYBR Green kit	ThermoFisher	F416L
RIPA buffer	Sigma	R0278-50M
Pierce protease inhibitor tablets	ThermoFisher	A32965
NuPAGE Sample Buffer (4x)	Life Technologies	NP0007
Bolt Bis-Tris Plus 4–12% 12 Well Gel	Life Technologies	NW04122BOX
Bradford BCA kit	ThermoFisher	PI23227
Nitrocellulose membrane	BioRad	1704270
Bio-Rad Trans-Blot Turbo	BioRad	1704150
Intercept (PBS) Blocking Buffer	LI-COR	927–90003
IRDye 800CW Donkey anti-Rabbit IgG	LI-COR	926–32213

### Biological resources

*Cell lines:*

- K562 CRISPRi cells from (38,39).
- HeLa CRISPRi cells from (40).
- Jurkat CRISPRi cells (clone NH7) were obtained from the Berkeley Cell Culture Facility (41).
- HEK293T cells from (38–40).

*Plasmids*:

- pMH0001 UCOE-SFFV-dCas9-BFP-KRAB (Addgene #85969);
- pCRISPRia-v2 (Addgene #84832)
- pLG_GI3 hU6 sgRNA vector (Addgene #111594)

### Data availability/sequence data resources

Sequencing data is available at GEO under accession number GSE207330. Processed RNA-seq data are provided in the supplementary materials.

### Data availability/novel programs, software, algorithms

For CRISPRi screen processing, sequencing data were aligned to the top 5 hCRISPRi-v2 library and quantified using the ScreenProcessing pipeline described in (42) with code available at (https://github.com/mhorlbeck/ScreenProcessing).

### Websites/database referencing

Molecular signatures database (https://www.gsea-msigdb.org/gsea/msigdb/).

Gene Ontology (GO) Resource (http://geneontology.org/).

### Media formulations

K562 and Jurkat cells were cultured in RPMI (Thermo Fisher Scientific, cat. 22400105) + 10% FBS (VWR, cat. 97068-085, lot 043K20) + 1× penicillin–streptomycin–glutamine (Gibco, cat. 10378016). HEK293T and HeLa cells were cultured in DMEM (Thermo Fisher Scientific, cat. 11965118) + 10% FBS + 1× penicillin–streptomycin–glutamine.

### Reporter cell line construction

A series of fluorescence reporters for monitoring the production of GFP driven by a near-cognate start codons was constructed based on a lentiviral expression plasmid backbone described in (38) (Addgene #85969). The vector contains an SFFV promoter, which drives strong expression and also contains no AUG sequences in the 5'UTR. Furthermore, a universal chromatin opening element (UCOE) upstream of the promoter prevents epigenetic silencing and a WPRE at the 3' end of the reporter sequence promotes transcript stability without causing premature termination during lentivirus transcription. The original dCas9-BFP-KRAB cassette was replaced with reporter elements consisting of superfolder GFP coded with a near-cognate start codon and mCherry driven by the EMCV IRES. Reporter elements were inserted via restriction digest of the original vector with MluI and NotI and Gibson assembly (New England Biolabs, cat. E2621X). Reporter variants with AUG, CUG, GUG or other near-cognate start codons were produced by PCR amplification of the GFP/IRES/mCherry cassette with unique 5′ primers with constant overhangs to allow for Gibson assembly (Supplementary Table S1).

Lentivirus for each fluorescence reporter was transduced into K562 cells for stable polyclonal expression at MOI < 1. Monoclonal cell lines were isolated for the CUG reporter variant by sorting on a Sony MA900 but provided only a small advantage in terms of the covariance of GFP and mCherry expression – thus polyclonal sorted populations were constructed to prevent experimental artefacts that might arise from a single cell bottleneck.

### Lentivirus production for CRISPRi screening

Lentivirus containing the hCRISPRi-v2 genome-wide CRISPRi sgRNA library was produced in thirteen 15 cm petri dishes of HEK293T cells. Prior to transfection, HEK293T cells were maintained at < 70% confluence during expansion. One day prior to transfection, cells were seeded at a density of 30 000 cells/cm^2^ such that they reached a confluence of ∼60–70% on the day of transfection. For transfection, each 15 cm dish of HEK293Ts was transduced with 20 μg sgRNA library, 6.75 μg of standard packaging plasmids v3 (for expression of VSV-G, Gag/Pol, Rev and Tat), and 81 μl Mirus transfection reagent (VWR, cat. 10767-122) in Opti-MEM. 24 h post-transfection, media was changed and supplemented with 1x ViralBoost (Alstem, cat. VB100). Supernatant containing lentivirus was harvested at 48 hours post-transfection. Cells were removed by centrifugation at 500g for 2 min and supernatant was filtered through a 0.45 μm filter (Millipore Sigma, cat. SLHP033RS) and frozen at -80°C. Lentivirus was then titered via a dilution series in K562s based on BFP expression at day 3 post-infection.

### CRISPRi screening

K562 cells were expanded into six T-175 flasks with 70 ml media per flask. Cells were split each day during expansion to 400 000 cells/ml, such that after 24 h of growth they reached a density of roughly 800 000 cells/ml. Cell counts and viability were evaluated by flow cytometry on an BD Accuri C6 Plus flow cytometer and viability was maintained at >90% prior to screening. To infect the K562 CRISPRi cells with the hCRISPRi-v2 sgRNA library (top 5 sgRNAs for each gene), cells were spinfected for 2 h to enhance infection efficiency. Briefly, 400M initial cells were pelleted by centrifugation at 200g for 5 min. Cells were resuspended in a total of 96 ml of fresh RPMI media + lentivirus + 8 μg/ml polybrene (Millipore Sigma, cat. 107689). Lentivirus volume was chosen based on prior titration curves with a target infection rate of 30%. The cell suspension was then aliquoted into eight 6-well plates with 2 ml per well and centrifuged at 1000g for 2 h at 37°C in a Sorvall Legend XTR centrifuge. After spinfection, cells were manually recovered by pipetting the contents each 6-well plate into three 50mL conical tubes and using 1mL of fresh media to wash each well and enhance the fraction of cells recovered. In practice, ∼80% of cells or 320M in total were recovered. Cells were again pelleted at 200g for 5 min. Lastly, cells were resuspended in fresh media at a density of 400 000 cells/ml and transferred to a 1 L spinner flask (DWK Life Sciences, cat. 356884) with a magnetic stir bar.

Twenty four hours after infection, cells were split into two biological replicates at a density of 400 000 cells/ml. At 48 h post-infection, cells were evaluated for percent infected by measuring the fraction of BFP + cells by flow cytometry. In practice, a ∼20% infection rate was achieved corresponding to a library coverage of ∼640×. At 48 h post-infection, cells were split to a density of 600 000 cells/ml in a total volume of 800 ml and 1 ug/ml of puromycin (Goldbio, cat. *P*-600-100) was added to select for sgRNA expressing cells. At day 3 post-infection, cells were again split to a density of 600 000 cells/ml and another 1 ug/ml of fresh puromycin was added. At day 3, roughly 30% of cells were BFP+/sgRNA + by flow cytometry. At day 4 and day 5 post-infection, cells were split into fresh media at 400 000 cells/ml with a total volume of 600 ml to permit recovery from puromycin selection. By day 5, ∼90% of cells were BFP+/sgRNA+.

Cells were sorted on day 6 post-infection on a BD FACS Aria II. For each round of sorting, 40M cells were gently pelleted at 200g for 4 min to help remove cell debris from puromycin selection. To enable a high sort rate, cells were resuspended in 1 ml of fresh media (∼40M cells/ml) and filtered through a mesh cap tube (Corning, cat. 352235) to disaggregate cell clumps. Cells were then placed on ice while awaiting flow sorting for a maximum of 2 h. Cells were flowed at a flow rate of 8, which achieved up to 25 000 events/second. Cells were sorted based on a cell viability gate (FSC versus SSC), a cell doublet gate (FSC-A versus FSC-H), an sgRNA expression gate (BFP+), and a GFP/mCherry ratiometric gate (top 15% and bottom 15% GFP/mCherry). In practice, ∼50–60% of cells passed the cell viability and singlet gates and ∼90% of cells were BFP+. Cells were sorted using custom sort setting with yield mask = 0 (ensuring deflection of only 1 drop and not adjacent drops) and purity mask = 8 (rejecting drops if a non-targeted particle falls within 4/32 of the leading or trailing drop). With these settings, ∼60% sort efficiency was achieved at flow rates of 20000–25000 events/second. In practice, ∼1000 cells/second were sorted into both the low and high GFP/mCherry populations. 12–20M cells were sorted over the course of 4 hours for each replicate. Sorted cells were then harvested by centrifugation and pellets were snap-frozen and stored at −80°C.

Genomic DNA was isolated from cell pellets with the Macherey-Nagel NucleoSpin Blood XL kit (Thermo Fisher Scientific, cat. NC1105387). Genomic DNA was isolated in a PCR free clean room and a small aliquot was quantified by NanoDrop, with ∼2.5 mg of total yield per pellet. 100 μl PCR reactions with 10 μg genomic DNA template each were set up in 96-well plates, using NEBNext Ultra II Q5 Master Mix (Thermo Fisher Scientific, cat. 50-105-0634). Unique Illumina TruSeq indices were incorporated for each sample. All PCR reactions from each sample were then pooled and 100 μl of the pool was size selected by double-sided SPRIselect magnetic bead clean-up (Thermo Fisher Scientific, cat. NC0406407). Libraries were quantified and sequenced by Illumina HiSeq 4000 SE50.

### Data analysis for primary CRISPRi screen

Sequencing data were aligned to the top 5 hCRISPRi-v2 library and quantified using the ScreenProcessing pipeline (https://github.com/mhorlbeck/ScreenProcessing) (42). sgRNA counts for the top 15% sample were divided by sgRNA counts for the bottom 15% sample and log_2_ transformed into a log_2_ enrichment score. An enrichment score for each gene was calculated by taking the mean of the top three sgRNAs targeting the gene. Significance at the gene level was calculated as Mann–Whitney *P*-value of the five sgRNAs targeting the gene compared to the set of 1895 non-targeting sgRNAs. Enrichment scores from the two replicates were averaged, while *P*-values were combined using Fisher's combined probability test.

**Figure 1.**
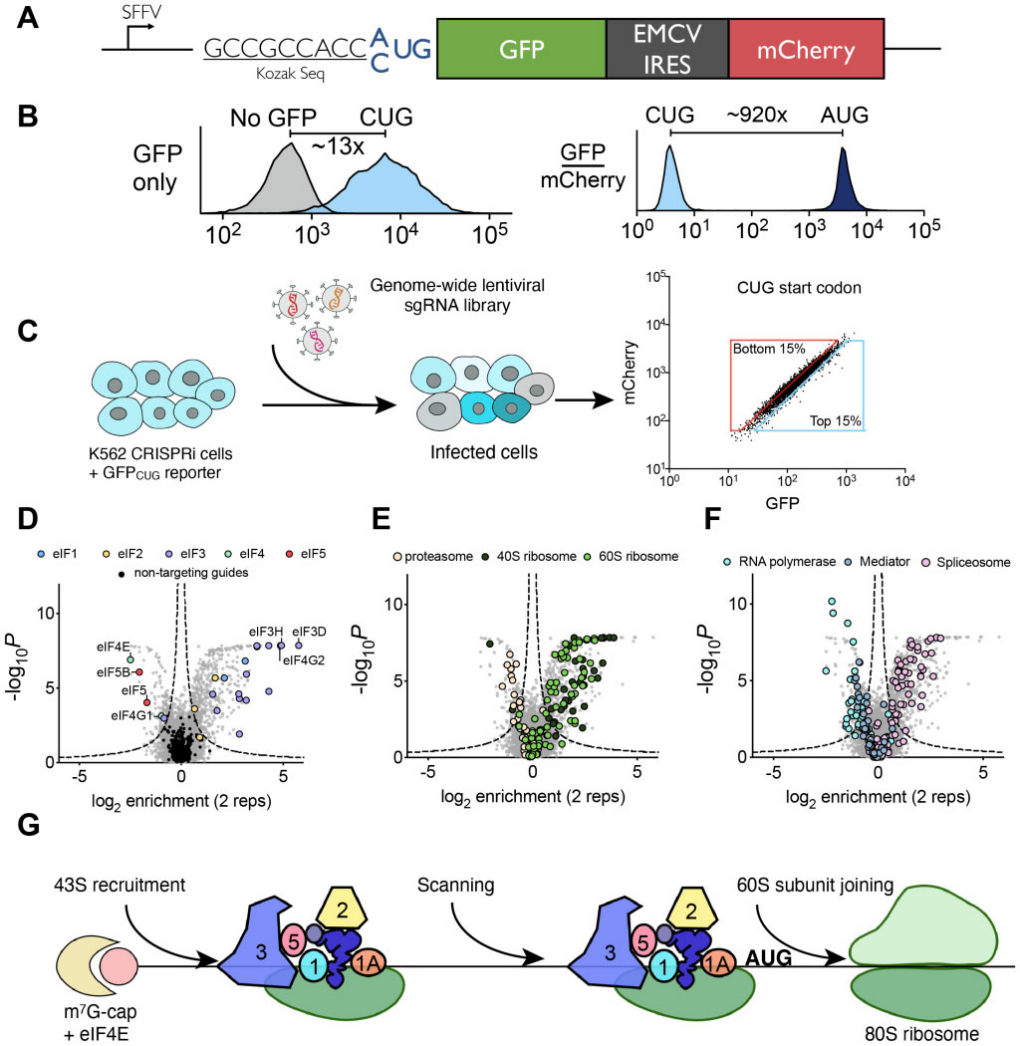
Genome-wide CRISPRi screens using a CUG translation reporter. (**A**) Schematic for lentiviral dual-fluorescence reporter to measure near-cognate start codon translation from a CUG start codon. (**B**) Comparative expression levels of reporter variants driven by CUG versus AUG start codons. (**C**) Workflow for FACS-based genome-wide CRISPRi screening in K562 cells. (**D**) Volcano plot of sgRNA enrichment scores for known initiation factors. (**E**) Volcano plot of sgRNA enrichment scores for proteosome or ribosome components. (**F**) Volcano plot of sgRNA enrichment scores for RNA polymerase, mediator, or spliceosome components. (**G**) Simplified cartoon of major steps in translation initiation, adapted from (45). Numbers are abbreviations for initiation factors, e.g. eIF3 is abbreviated as 3.

Enriched and depleted gene sets were defined based on an empirically derived threshold based on the product of the enrichment score × −log_10_*P*-value. The threshold was chosen such that no negative control sgRNAs met the threshold. GO analysis was performed on enriched and depleted gene sets (http://geneontology.org/) and genes belonging to major GO categories were visualized via volcano scatter plots (Figure 1D–F).

### Secondary screening sgRNAs

For individual evaluation and re-testing of sgRNA phenotypes, 96 sgRNA expression plasmids were cloned in arrayed format. sgRNA protospacers for each target gene were inserted by annealing complementary synthetic oligonucleotide pairs (Integrated DNA Technologies) with BstXI and BlpI restriction site overhangs and ligation into BstXI/BlpI digested pCRISPRia-v2 (marked with a puromycin resistance cassette and BFP, Addgene #84832) (42). To promote annealing, the two oligos were added to 1x duplex buffer (Integrated DNA Technologies) at a final concentration of 2 μM, heated to 95°C on a PCR block for 5 min, and slowly cooled to room temperature. Oligos were then diluted 1:40 in 1× duplex buffer and added to a ligation reaction with 1 μl cut plasmid (25 ng/μl), 1 μl diluted oligos, 0.5 μl fresh 10× T4 ligase buffer (with limited freeze thaw cycles), 0.5 T4 ligase (New England Biolabs, cat. M0202S), and 2 μl water. Ligation was performed at RT for 1 h and 1 μl of ligation product was transformed into 10 μl of Stellar Competent Cells (Takara Bio, cat. 636766). Protospacer sequences used for individual sgRNAs are listed in Supplementary Table S1.

Individual sgRNA expression plasmids were transfected into HEK293T cells for lentivirus production in arrayed format in 6-well plates. Lentiviruses were then transduced into reporter cell lines by spinfection for K562 cells and Jurkat cells or reverse transduction for HeLa cells, typically in 24-well format. Reporter cells were then evaluated by flow cytometry at day 5 post-transduction to allow for depletion of sgRNA target genes. MOI was typically <1, resulting in ∼15–30% of cells infected. Flow cytometry analysis was then performed using uninfected cells as an internal control for each well and reporter phenotypes were quantified as the difference in GFP (ΔGFP) and mCherry (ΔmCherry) between sgRNA-infected and uninfected cells within the same well.

### Double knockdown sgRNAs

Dual sgRNA expression vectors were cloned in accordance with the method previously described in (43). Each of 24 sgRNA protospacers (Supplementary Table S1) were cloned into a variant of the single sgRNA plasmid with a modified human U6 promoter replacing the original mouse U6 promoter (Addgene #111594). After verification by Sanger sequencing, a fragment containing the human U6 and sgRNA components was PCR amplified and Gibson cloned into the XhoI restriction site of an original single sgRNA expression plasmid with sgRNAs targeting eIF3D, eIF4G2, eIF5 or a non-targeting sgRNA. As with single sgRNA expression vectors, dual expression vectors were transfected into HEK293T cells for lentivirus production in arrayed format and transduced into K562 reporter cells. Reporter phenotypes were analyzed by internally controlled comparisons to uninfected cells within the same well.

### Western blots

K562 cells were infected with lentiviral constructs containing sgRNAs targeting eIF1A, eIF2α, eIF3A, eIF3D, eIF3G, eIF3H, eIF3M, eIF4G2 and eIF5. Forty-eight hours post-infection, 2 μg/ml puromycin was added to RPMI media to select for cells expressing sgRNA. Cells were spun down at 72 h post-infection at 200g for 2 min to help remove debris and dying cells. The pellet was then resuspended in fresh media with 2 μg/ml puromycin for an additional day. Cells were recovered in normal growth media from 4 days post-infection to 5 days post-infection. sgRNA containing cells were sorted to purity on a Sony MA900 cell sorter at 5 days post-infection and pellets were immediately lysed in 30 μl ice-cold RIPA buffer + protease inhibitor (ThermoFisher, cat. A32965) per 1 million cells. After 30 min of incubation in lysis buffer at 4°C, cells were centrifuged at 16 000g for 5 min at 4°C. Supernatant was collected and snap frozen in liquid nitrogen and stored at −80°C.

Protein concentrations in each lysate were quantified using a Bradford BCA kit (ThermoFisher, cat. PI23227) Lysate was normalized to 1 μg/μl in RIPA buffer. 30 μl of lysate was added to 10 μl of NuPage Sample Buffer (4×), heated to 70°C on a PCR thermocycler, and loaded onto a Bolt 4–12% polyacrylamide gel (ThermoFisher, NW04122BOX). Four replicate gels were run for 45 min at 165V in MOPS buffer to allow for blotting of multiple eIF3 subunits of similar molecular weight. Protein was then transferred onto a nitrocellulose membrane (BioRad, cat. 1704270) with a Bio-Rad Trans-Blot Turbo (BioRad, cat. 1704150). The membrane was blocked with Intercept (PBS) Blocking Buffer (LI-COR, cat. 927–90003) for 1 h at RT. Membrane was incubated overnight at 4°C with primary antibody, with catalog numbers and dilutions for each antibody listed in Supplementary Table S2. Membrane was washed 3× with TBST and incubated with secondary antibody (Licor IRDye 800CW Donkey anti-Rabbit IgG, cat. 926-32213) at 1:15000 dilution. Membrane was washed 3× with TBST and imaged on a LI-COR Odyssey CLX.

### Quantitative RT-PCR

K562 cells with sgRNAs targeting eIF1A, eIF2α and eIF5 were grown in parallel with cells grown for western blotting (see above). Cells were puromycin selected and 200 000 cells were sorted at 5 days post-infection. Cell pellets were immediately lysed in RNAse-free Trizol and stored at −80°C. RNA was extracted with a Direct-zol RNA miniprep kit (Zymo Research, cat. R2051). RNA was reverse transcribed with SuperScript IV VILO (ThermoFisher, cat. 11756050), and cDNA was amplified with the DyNAmo ColorFlash SYBR Green kit (ThermoFisher, cat. F416L). Primers for GAPDH were used as loading controls and no-RT controls were performed to control for genomic DNA contamination. Amplifications were performed in duplicate and quantified on a QuantStudio Flex 7 Real-Time PCR system in 96-well plates.

### eIF3D structure function variants

A donor plasmid containing a cDNA-based eIF3D ORF was ordered from the Harvard CCSB Human ORFeome collection (no longer operational) (BC080515, Internal ID 55224). The eIF3D ORF was cloned into the single sgRNA vector containing an sgRNA targeted against the endogenous copy of eIF3D. As the sgRNA targets the endogenous promoter, it does not target the SFFV promoter that drives the expression of exogenous eIF3D. The original eIF3D sgRNA plasmid was digested with NheI and EcoRI. The eIF3D ORF was then inserted downstream of Puro-T2A-BFP via three-piece Gibson Assembly, with one PCR fragment reconstituting the Puro-T2A-BFP cassette and one PCR fragment consisting of the eIF3D ORF with an upstream P2A to maintain expression on the same transcript. eIF3D variants were constructed via primers that resulted in N-terminal truncation, C-terminal truncation, modification of phosphorylation sites proximal to the C-terminus, or triple mutants in helix α5 or α11. Lentivirus for each variant was transduced into K562 reporter cells. Cells expressing each construct were gated by BFP expression and compared to cells without BFP expression. Reporter phenotypes were quantified by measuring changes in GFP and mCherry expression.

### Overexpression plasmids

Donor plasmids containing cDNA-based ORFs for cJun, ABCD1, eIF2A, eIF2D, MCTS1 and DENR were obtained from the ORFeome Collaboration Clones (Horizon Discovery). Each ORF was cloned into the eIF3D sgRNA vector and an otherwise identical vector containing a non-targeting sgRNA. As with the eIF3D ORF, the ORFs were inserted downstream of Puro-T2A-BFP-P2A. Lentivirus for each overexpression construct was transduced into K562 reporter cells. Cells expressing each construct were gated by BFP expression and compared to cells without BFP expression. Reporter phenotypes were quantified by measuring changes in GFP and mCherry expression.

### Flow cytometry

Data were collected on an Attune NxT flow cytometer (Thermo Fisher Scientific) and analyzed with custom Matlab scripts. Viable cells were gated based on forward and side scatter with a manually drawn gates. Doublets were filtered based on FSC-A and FSC-H with manually drawn gates. Both viable cell and doublet filters were applied to all cells within a well. Next, sgRNA containing cells were distinguished from uninfected cells by BFP expression with a linear gate. Mean GFP and mCherry expression was then calculated for sgRNA expressing cells and uninfected cells. The difference in GFP expression (ΔGFP) between the two populations was then calculated, representing the change in near-cognate start codon translation. The difference in mCherry expression (ΔmCherry) was also calculated, representing a change in IRES-driven translation. To quantify a normalized reporter score, differences in mCherry were subtracted from differences in GFP and log_2_ transformed. Thus, log_2_(ΔGFP − ΔmCherry), was used as the primary metric for the effect of an sgRNA, dual sgRNA, or overexpression construct on the reporter.

### Bulk RNA-seq

K562, Jurkat, and HeLa cells were infected with individual sgRNAs targeting eIF3D. K562 cells were also infected with individual sgRNAs targeting eIF4G2, eIF1A or ZNF324. Cells were then expanded for 5 days. At day 5, ∼1M sgRNA expressing cells were sorted on a Sony MA900 cell sorter based on BFP + expression. Cells were then pelleted, snap-frozen and stored at −80°C. High quality RNA was extracted by adding RNAse-free Trizol (Thermo Fisher Scientific, cat. 15596026) to each pellet and processing with the Zymo Research Direct-zol RNA miniprep kit (Zymo Research, cat. R2050). RNA-seq was performed using the Illumina TruSeq Stranded Total RNA kit (Illumina, cat. 20020599) according to the manufacturer's instructions, with the exception of the final PCR step for which only 10 cycles were used to prevent overamplification. The final pooled library was sequenced with 50 bp single end reads on a HiSeq 2500.

RNA-seq sequencing reads were aligned to hg19/GRCh37 with STAR aligner and quantified with featureCounts. Fold-changes were calculated by comparison of counts between wild-type cells and cells expressing sgRNAs for given target genes. Transcriptional responses were then compared to annotated gene sets from the Molecular Signatures Database (MSigDB).

### Perturb-seq analysis

eIF3D did not cluster with any other genes in the original Perturb-seq analysis (44), as clustering was performed in a robust fashion that only identified the strongest clusters. In this original analysis, not all genes were members of transcriptional clusters. However, the sgRNA targeting eIF3D produced a strong transcriptional phenotype as measured by the number of differentially regulated genes. We thus performed a more permissive clustering on a high-dimensional (20 dimensions) embedding of the data. The embedding in this case served as a light imputation step that potentially caused perturbations to be drawn closer to their presumptive relatives. This analysis produced a clustering visualization akin to the one presented in (44). With this analysis, eIF3D knockdown cells clustered with cells depleted for eIF3E/F/H/L/M, eIF4A1, eIF4G2, eIF1A, DDX3X, CSDE1, STRAP and ZNF324.

The clustering method did not inherently identify genes that were most responsible for distinguishing the eIF3D cluster from all other clusters. Thus, we rationally picked comparison gene sets likely to influence translation or activate NF-κB. These comparison sets consisted of all other known initiation factors, ribosomal proteins from the small or large subunit, and genes known to activate NF-κB upon knockdown. To visualize the differential transcriptional response of each gene set, we filtered the response by genes with maximum normalized *z*-score >1 (absolute value) and median normalized *z*-score >0.1 (absolute value) across all genes. sgRNA target genes and transcriptional responses were then clustered by k-means clustering with five clusters randomly seeded for the sgRNA target genes and three clusters seeded for the transcriptional response. Genes in the transcriptional response clusters were evaluated by GO enrichment and the top GO terms were empirically summarized into cluster labels. The transcriptional response clusters tended to contain several broad categories of genes, with the exception of ribosomal proteins.

## RESULTS

### Genome-wide CRISPRi screens identify candidate regulators of alternative start codon usage

To enable systematic genetic screening approaches, we designed a series of translational fidelity reporters. Our most basic reporter design utilized a bicistronic fluorescence protein construct: superfolder GFP encoded with a CUG near-cognate start codon and mCherry driven by the EMCV internal ribosome entry site (IRES) (Figure 1A). We chose CUG because it is the most frequently utilized near-cognate start codon, while the IRES/mCherry element acted as an internal expression control. To minimize translation initiation at other sites within the 5'UTR, we removed all AUG sequences upstream of the CUG start codon. We observed that compared to a standard AUG start codon, a CUG start codon with a consensus Kozak sequence produced ∼920x lower levels of GFP expression (Figure 1B, normalized to mCherry). However, due to the presence of a strong SFFV promoter, the CUG start codon reporter produced ∼13× higher levels of GFP fluorescence compared to the background autofluorescence of wild-type K562s. To quantify changes in the rate of GFP translation, we normalized total GFP expression by mCherry expression. This ratiometric approach allowed us to account for changes in transcript levels, which would exert equivalent effects on both GFP and mCherry. In addition, we observed a high degree of correlation (Pearson's correlation *r* = 0.96) between GFP and mCherry expression across a polyclonal population of cells, which helped to control for variation in absolute GFP expression levels due to factors such as cell size and lentiviral integration site. The coefficient of variation (CV) for the GFP/mCherry ratio was 22%, whereas GFP alone had a CV of 76%.

Next, we leveraged the low degree of variation and noise in our fluorescence reporter for large-scale FACS-based screening. To identify genetic perturbations that would alter the frequency of CUG start codon initiation, we infected our reporter cell line with a genome-wide lentiviral hCRISPRi-v2 sgRNA library (42). After 6 days of selection and outgrowth to ensure adequate recovery and knockdown, we sorted cells with high or low GFP/mCherry ratios. Gates for the top 15% and bottom 15% of cells differed by only ∼46%, in theory allowing our screen to distinguish sgRNAs with ∼2-fold effects on CUG initiation rates (Figure 1C). Lastly, we used next-generation sequencing to quantify the sgRNAs present in the high vs. low GFP/mCherry subpopulations.

Our screen revealed strong enrichment of sgRNAs targeting known initiation factor complexes. sgRNAs targeting eIF5/5B, eIF4G1 or eIF4E were enriched in the low GFP/mCherry population while sgRNAs targeting eIF1/1A, eIF2, eIF3 or eIF4G2 were enriched in the high GFP/mCherry population (Figure 1D). In addition, components of the spliceosome and ribosome were enriched in the high GFP/mCherry population whereas members of the proteasome, RNA polymerase, and mediator complexes were depleted (Figure 1E, F). In total, we observed 476 candidate genes whose knockdown increased the ratio of GFP to mCherry and 154 candidate genes that decreased the ratio (Supplementary Table S3). The majority of factors identified in our screen were highly essential, demonstrating the utility of our CRISPRi screening approach in evaluating factors involved in essential cell biological processes (Supplementary Figure S1A). However, the majority of essential genes (1923/2324) had no effect on our fluorescence reporter. In addition, the ability of essential genes to either increase or decrease GFP/mCherry suggested that simple impairment of cellular growth was not responsible for changes in reporter expression.

### Secondary screening decouples alternative start codon translation from IRES-dependent translation

**Figure 2.**
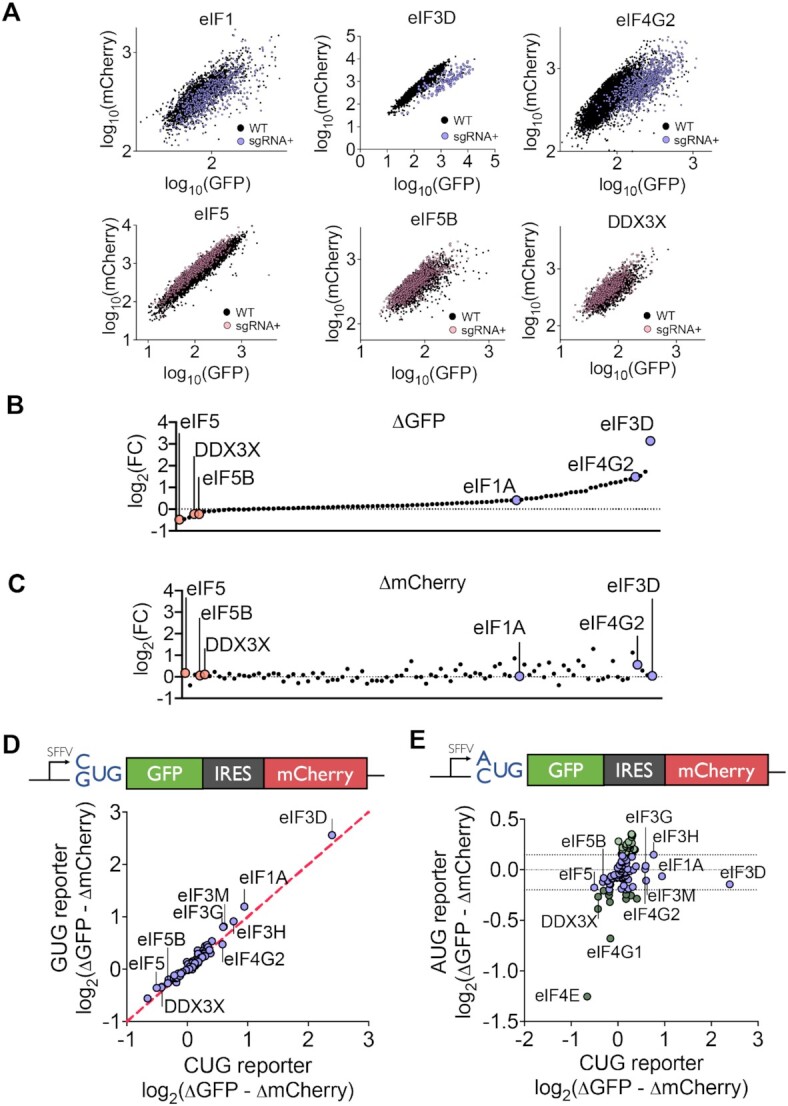
Validation of individual sgRNA phenotypes across reporter variants. (**A**) GFP vs. mCherry expression for co-cultures between wild-type cells and sgRNA containing cells. (**B**) Change in CUG-driven GFP expression upon sgRNA knockdown for 96 individual sgRNAs. (**C**) Change in IRES-driven mCherry expression upon sgRNA knockdown for 96 individual sgRNAs. (**D**) Comparison of sgRNA depletion phenotypes in cells expressing a CUG translation reporter versus GUG translation reporter. (**E**) Comparison of sgRNA phenotypes in cells expressing a CUG translation reporter versus AUG translation reporter.

Next, we individually retested 96 candidate sgRNAs targeting 96 genes to distinguish increases in GFP translation at the CUG start codon from reductions in IRES-mediated mCherry translation, as both effects would similarly alter the GFP to mCherry ratio (Supplementary Table S4). To do so, we performed sgRNA-mediated knockdowns in internally controlled co-cultures, with uninfected wild-type cells (BFP−) and sgRNA-expressing cells (BFP+) mixed within the same well. As each well contained only a single sgRNA, we decoupled GFP and mCherry fluorescence into average GFP and average mCherry expression levels across a population of cells (Figure 2A). For each sgRNA, we quantified whether the change in GFP/mCherry ratio could be attributed to changes in GFP levels or in mCherry levels. sgRNAs targeting known initiation factors elicited larger changes in GFP levels (Figure 2B) compared to mCherry levels (Figure 2C). By contrast, sgRNAs targeting ribosomal small subunit proteins and spliceosome factors primarily altered the mCherry levels (Supplementary Figure S1B). Overall, 43/96 sgRNAs tested exhibited stronger effects on IRES-mediated translation compared to CUG translation – thus a substantial number of hits from the primary screen were likely due to trans-factors required by the EMCV IRES. In theory, some of these false positive candidates could have been mitigated in primary screening via usage the type 4 CRPV IRES, which directly recruits the 40S and does not depend on initiation factors. However, a false negative result would require a factor to change GFP and mCherry levels by the same amount, and the sensitivity of our assay allowed us to recover candidate genes with quantitatively different effects on CUG and IRES-mediated translation. Furthermore, results from our secondary screening allowed us to exclude factors that primarily affect IRES-mediated translation from further follow-up experiments.

We then explored whether each of our candidate genes specifically modulated initiation at CUG start codons or at near-cognate start codons more broadly. To do so, we replaced the CUG start codon in our bicistronic fluorescence reporter with a GUG start codon, the second most frequently used near-cognate start codon. Overall GFP expression from the GUG reporter was comparable to that from the CUG reporter (Supplementary Figure S1C). Across all 96 candidate sgRNAs, changes in alternative start codon usage were virtually identical for the two reporters (Pearson's correlation *r* = 0.98) (Figure 2D).

As an additional control, we tested the effect of the knockdowns in reporter cells expressing GFP from a standard AUG start codon. GFP expression was restored to high levels in the AUG reporter, while mCherry levels remained unchanged. We observed that both CUG and AUG reporters exhibited decreased GFP levels upon knockdown of eIF4E or eIF4G1, as both are key factors in the initial recruitment of the 40S small subunit to mRNA, but not in start codon discrimination (Figure 2E). In contrast, sgRNAs targeting eIF3 subunits, eIF4G2 or eIF1/1A increased expression of the CUG reporter but not of the AUG reporter. Conversely, sgRNAs targeting eIF5/5B reduced translation from the CUG near-cognate reporter but not the AUG reporter. These results were consistent with previous screens in yeast that identified eIF1/1A and eIF5/5B as modifiers of start codon stringency (26,29,32). In addition, subsequent studies have shown that eIF1A, eIF5 and eIF5B are key factors in catalyzing the formation of the 80S ribosome, the final step in start codon recognition (8,45). However, the roles of eIF3 and eIF4G2 in alternative start codon usage have not been previously described.

To further exclude the possibility that the effects of our knockdowns were mediated by interactions with the EMCV IRES, we constructed reporter cell lines with no IRES elements. To maintain the normalization properties of a dual-fluorescence reporter, we integrated a CUG-encoded GFP and an AUG-encoded mCherry via separate lentiviral vectors and isolated a monoclonal reporter cell line. We confirmed that this no-IRES reporter exhibited highly concordant phenotypes across most candidate knockdowns, with the exception of IRES-dependent candidates that were also identified during the initial round of validation (Pearson's correlation *r* = 0.76) (Supplementary Figure S2A). In concordance with previous control experiments, we observed that depletion of eIF3 components, eIF4G2, and eIF1A resulted in increased translation from alternative start codons whereas eIF5 knockdown repressed alternative start codon usage. Lastly, to control for specific RNA binding motifs in the 5′UTR, we replaced the SFFV promoter with an EF-1α promoter and verified that eIF3D knockdown promoted increased alternative start codon usage (Supplementary Figure S2B).

### eIF3 plays a major role in start codon discrimination

**Figure 3.**
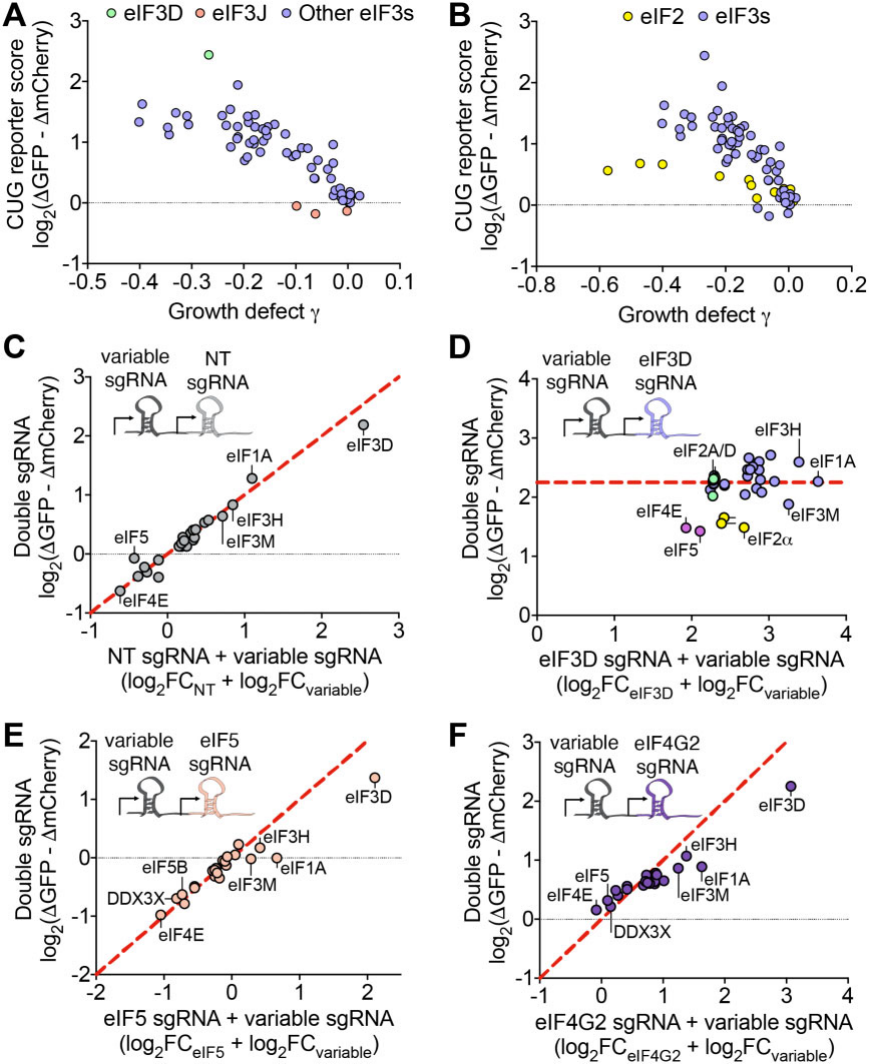
eIF3D exerts a dominant effect on start codon selection. (**A**) Scatter plot of CUG reporter score (log_2_(ΔGFP – ΔmCherry)) versus growth defect (log_2_ sgRNA depletion per doubling) for sgRNAs targeting subunits of eIF3. (**B**) Scatter plot of CUG translation phenotype for sgRNAs targeting subunits of eIF3 and sgRNAs targeting subunits of eIF2. (**C**) Double sgRNA knockdowns with 24 candidate sgRNAs and a non-targeting sgRNA, (**D**) an sgRNA targeting eIF3D, (**E**) an sgRNA targeting eIF5, (**F**) or an sgRNA targeting eIF4G2.

We next investigated the effects of eIF3 depletion on alternative start codon usage, as four out of the six strongest sgRNAs from our validation screening targeted subunits of eIF3 (eIF3D, eIF3G, eIF3H and eIF3M). We first asked whether depletion of these four subunits uniquely promoted CUG start codon usage compared to other eIF3 subunits. We hypothesized that not all sgRNAs targeting eIF3 would be fully active, as some fraction of sgRNAs would not achieve sufficient depletion of their target genes. Indeed, several lines of evidence revealed that sgRNA-mediated depletion was highly variable and incomplete, which was likely due to the strong essentiality of eIF3 components. First, we observed depletion of any eIF3 subunit other than eIF3J resulted in enhanced translation of the CUG near-cognate start codon reporter (Figure 3A). Secondly, we observed that the growth defects induced by sgRNAs targeting eIF3 subunits were strongly correlated to the magnitude of reporter phenotypes. The sgRNAs with the strongest growth defects were comparable to sgRNAs targeting ribosomal subunits (Supplementary Figure S3A). Lastly, we directly measured the extent of sgRNA-mediated depletion by Western blot for individual sgRNAs targeting eIF3A, eIF3D, eIF3G, eIF3H and eIF3M (Supplementary Figure S3B–E, Supplementary Table S2). Knockdown efficiency was poor for eIF3A, with only 25% depletion. Depletion was intermediate for eIF3D, eIF3H, and eIF3M, ranging from 48% to 80%. Only eIF3G exhibited greater than 90% knockdown efficiency. These results suggest that individual subunits of the eIF3 complex may have distinct depletion thresholds, at which point the overall activity of the complex is impaired and cellular translation is disrupted.

We extended our comparison to other key initiation factors. Knockdowns of eIF2 produced equally severe growth defects as eIF3 depletion but comparatively milder increases in CUG start codon usage (Figure 3B). Measurements of sgRNA-mediated transcript depletion by qRT-PCR showed ∼73% knockdown efficiency (Supplementary Figure 3F). Meanwhile, eIF1 or eIF1A knockdown also elicited an increase in CUG translation, with depletion of eIF1 causing no impairment to growth (Supplementary Figure S4A). qRT-PCR on an eIF1A sgRNA revealed only 37% knockdown efficiency. Double knockdown of eIF1 and eIF1B, which encode the same polypeptide from separate genomic locations, resulted in a purely additive effect that remained weaker than knockdown of eIF3 subunits, indicating a lack of genetic buffering between these factors (Supplementary Figure S4B, C). These results demonstrated that our CRISPRi screening approach was capable of uncovering cellular phenotypes in highly essential genes whose complete knockouts were unviable. In addition, the variable activity of the five sgRNAs targeting each eIF3 and eIF2 subunit acted as a dose titration experiment, revealing a stronger relationship between the degree of growth impairment and the frequency of near-cognate start codon usage for eIF3.

To explore potential interactions effects between key initiation factors, we performed a set of targeted double-knockdown genetic interaction experiments. We selected a set of 24 potential interaction partners that included major initiation factor candidates, ribosomal subunits, and additional genes from our validation screens (Supplementary Tables S5 and S6). We cloned each sgRNA into a dual-sgRNA vector and verified that each interacting sgRNA maintained its original activity when paired with a non-targeting sgRNA (Figure 3C). Next, we introduced sgRNAs targeting eIF3D, eIF4G2, or eIF5 in combination with each potential interaction partner. We chose eIF3D as a representative of eIF3 due to its strong effect size and because of previous literature indicating that eIF3D depletion is unique among eIF3 subunits in preserving the structural integrity of the remaining eIF3 complex (46,47). In addition, eIF3D has recently been found to play a separate role in alternative cap-binding via a physical interaction with eIF4G2 (48–50). Under an additive model with no interactions, we would expect that the combined effect of each double knockdown to equal the sum of the two individual knockdowns. Instead, we observed that eIF3D knockdown exerted a maximal effect, with no other knockdowns significantly increasing the extent of CUG translation beyond eIF3D knockdown alone (Figure 3D). However, we observed that knockdowns of eIF2, eIF4E, and eIF5 reduced the effect of eIF3D knockdown. These data suggest that increased translation of the CUG reporter was largely mediated by standard eIF4E cap-dependent processes and eIF2-linked initiator methionine tRNA. By contrast, depletion of alternative eIF2 initiation factors eIF2A and eIF2D with two independent sgRNAs had no effect on the eIF3D depleted cells (Supplemental Figure S4D). These results indicate that initiation at the CUG start codon was not primarily driven by its cognate leucine tRNA (51,52). In addition, overexpression of alternative initiation factors and ribosome recycling factors ABCE1, eIF2A, eIF2D, MCTS1, or DENR had no effect on either wild-type cells or eIF3D depleted cells (Supplementary Figure S4E).

In contrast to eIF3D, double knockdowns with eIF5 conformed exactly to the additive expectation of independent sgRNAs. For nearly all potential interaction partners, double knockdowns with eIF5 exhibited decreased CUG translation compared to the single sgRNA knockdowns alone (Figure 3E). This lack of genetic interactions was consistent with the role of eIF5 in mediating the final steps of initiation, downstream of other potential factors. However, we observed that eIF5’s known interaction partner eIF1A deviated substantially from the additive expectation. As eIF5 and eIF1A physically interact and compete for binding to eIF5B (53–56), loss of eIF5 fully negated the eIF1A phenotype, despite the single sgRNA eIF1A phenotype being stronger than the single sgRNA eIF5 phenotype.

Lastly, we performed double knockdown experiments with eIF4G2. eIF4G2 is a homolog of eIF4G1 that cannot bind to eIF4E and instead relies on the cap-binding properties of eIF3D to recruit the ribosome to select mRNAs (48–50). eIF4G2 exhibited substantial genetic interactions with eIF3 subunits and eIF1A (Figure 3F). The combined knockdown phenotypes of eIF3H/eIF4G2 and eIF3M/eIF4G2 were buffered and remained substantially weaker than the effect of eIF3D knockdown alone. These data suggest that effects of eIF4G2 on near-cognate start codon usage could depend on its interactions with eIF3.

### eIF3D N-terminal domain is required for stringent start codon selection

**Figure 4.**
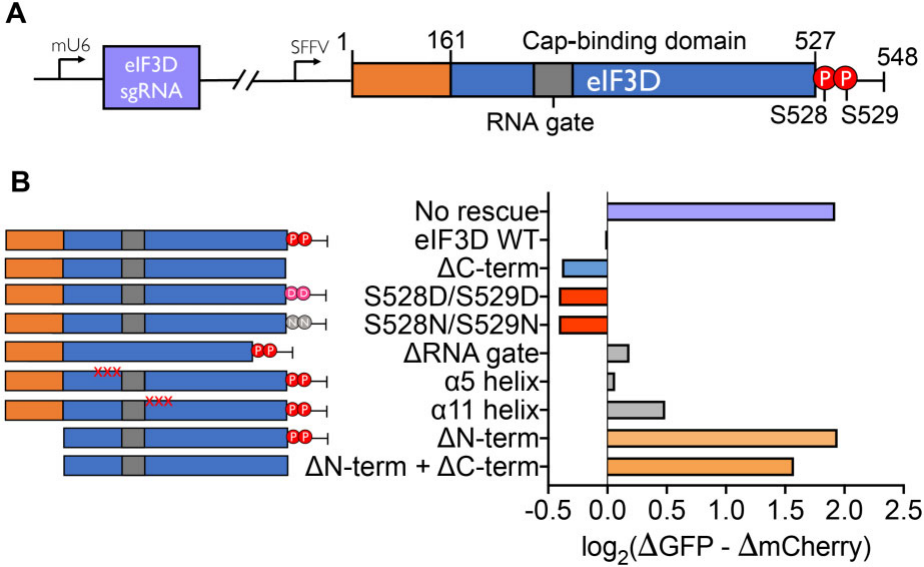
Evaluation of the structure-function relationship of eIF3D mutants. (**A**) Schematic of lentiviral construct for simultaneous knockdown of endogenous eIF3D and rescue with exogenous eIF3D mutants. (**B**) CUG reporter phenotypes for simultaneous eIF3D knockdown and rescue with eIF3D mutants.

To systematically explore the structure to function relationships within eIF3D, we combined sgRNA-mediated knockdown of endogenous eIF3D with exogenous rescue using various mutants (Figure 4A, B). Full length eIF3D overexpression fully rescued eIF3D knockdown. C-terminal truncation, which removed residues 527–548 and a long poly-glutamic acid tract, remained capable of rescuing start codon selectivity. Next, we tested serine to aspartic acid (S528D/S529D) and serine to asparagine (S528N/S529N) mutations at two casein kinase II (CK2) phosphorylation sites near the eIF3D cap-binding domain. Phosphorylation of these residues was recently reported to activate eIF3D in response to metabolic stress (49). However, neither the phospho-mimetic aspartic acid substitution nor the non-phosphorylatable asparagine had an effect on eIF3D rescue. We also tested removal of the ‘RNA gate’, an unstructured loop of 15 amino acids between strand β5 and helix α6. Previous studies showed that this loop structurally regulates the binding of eIF3D to mRNA and potentially prevents promiscuous mRNA binding (48). This mutant exhibited nearly full rescue as well, with only a minor increase in CUG translation. Structure-guided triple mutants of helix α5 or α11 that fully abolish eIF3D cap-binding activity also rescued depletion of endogenous eIF3D, indicating that the cap-binding activity of eIF3D was dispensable for near-cognate start codon usage. Finally, we tested an N-terminal truncation of residues 1–160. This N-terminal truncation mutant represents the minimal stable human cap-binding domain and was shown to bind to the *c-Jun* mRNA 5′ cap *in vitro* (48). Unlike other mutants we tested, the N-terminal truncation mutant was unable to rescue loss of endogenous eIF3. These data further suggest that rescuing the cap-binding function of eIF3D alone was not sufficient for restoring normal start codon initiation. Recent cryo-EM structures showed that the N-terminal tail of eIF3D interacts with eIF3E and eIF3C, suggesting that these interactions may be essential in connecting eIF3D to the core eIF3 complex, which may then collectively regulate near-cognate translation (57,58).

### Transcriptional signatures of eIF3D depletion in multiple cell types

**Figure 5.**
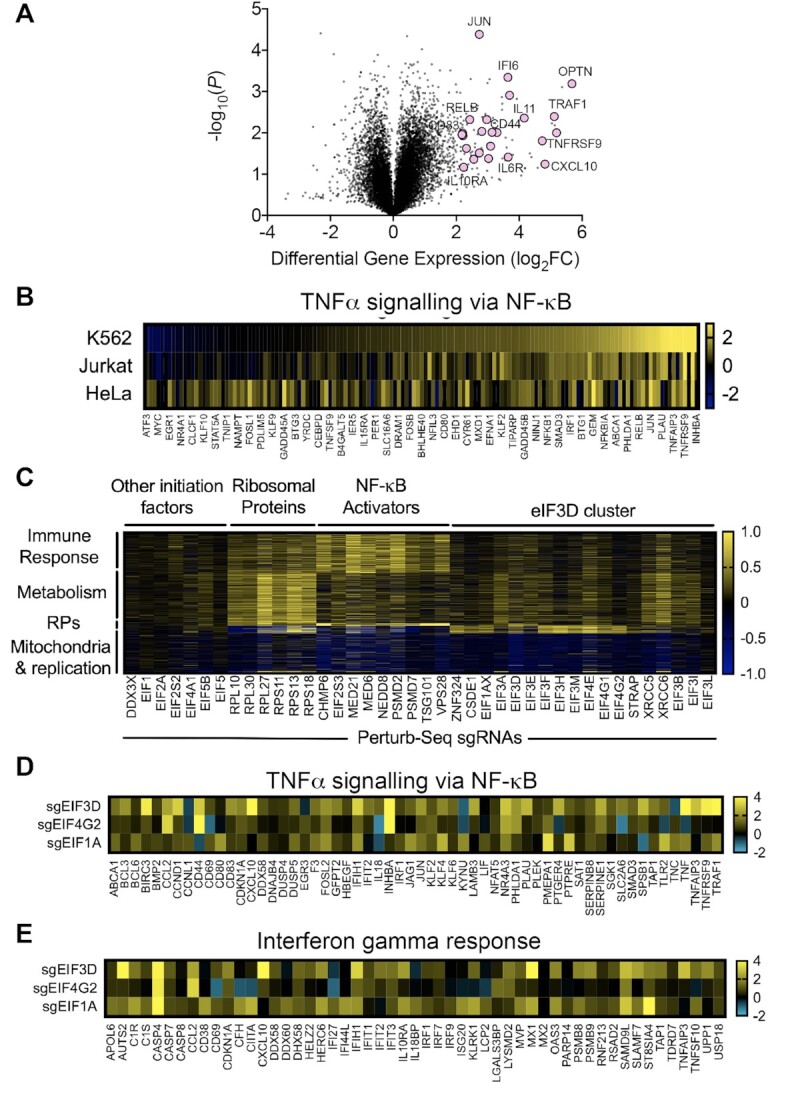
Transcriptional signatures of eIF3D depletion in multiple cell types. (**A**) Volcano plot of RNA-seq expression in K562s with eIF3D knockdown compared to wild-type. **(B**) RNA expression of genes annotated as TNFα signaling via NF-κB by the Molecular Signatures Database (MSigDB) in K562, HeLa, and Jurkat cells. (**C**) Genome-wide perturb-seq transcriptional profiles for eIF3D and closely clustered genes compared to control genes. (**D**) Bulk RNA-seq gene expression for genes annotated as TNFα signaling via NF-κB by the Molecular Signatures Database (MSigDB) in K562 cells depleted for eIF3D, eIF4G2, or eIF1A. (**E**) Bulk RNA-seq gene expression for genes annotated as interferon gamma response by the Molecular Signatures Database (MSigDB) in K562 cells depleted for eIF3D, eIF4G2 or eIF1A.

We performed bulk RNA-seq in K562, HeLa and Jurkat cells to determine the transcriptional signature of eIF3 depletion across cell types. RNA-seq in K562 cells depleted for eIF3D revealed strong upregulation of immune-related genes, including IL-8, IL-32, IFI6, CD83 and CD44 (Figure 5A, Supplementary Table S7). Comparison to the Molecular Signatures Database (MSigDB) revealed TNFα signaling via NF-κB as the dominant transcriptomic signature (*P* < 10^−17^), with 44/145 annotated genes upregulated by >2-fold (Figure 5B). This transcriptional signature was shared in eIF3D-depleted Jurkat and HeLa cells. Jurkat cells upregulated a highly similar subset of genes compared to K562s, while HeLa cells upregulated a separate subset of genes within the TNFα signaling via NF-κB annotation set (Figure 5B).

Next, we asked whether the transcriptional profile of eIF3D depletion was similar to any other genetic perturbation using data from genome-wide Perturb-seq in K562 cells (44). Using a permissive clustering scheme on a 20-dimensional embedding of the Perturb-seq data, we observed that eIF3D knockdown cells clustered with cells depleted for eIF3E/F/H/L/M, eIF4A1, eIF4G2, eIF1A, DDX3X, CSDE1, STRAP and ZNF324 (Figure 5C). The grouping of core eIF3 subunits with additional initiation factors demonstrates the power of Perturb-seq to identify functional modules based on shared changes in gene expression and mirrors known biochemical interactions. Among the remaining genes in the cluster, we determined that the major effects of the sgRNA targeting ZNF324 were mediated by off-target repression of eIF3H (Supplementary Figure S5A). Secondary screening suggested that CSDE1 and STRAP mainly affected IRES-mediated translation rather than near-cognate start codon usage (Supplementary Figure S5B-D).

We further analyzed the genome-wide Perturb-seq data to test whether any part of the eIF3D transcriptional signature was unique compared to all other genes. As a comparison set, we picked major classes of highly essential genes such as ribosomal proteins, other major initiation factors, and genes known to activate NF-κB such as members of the ESCRT complex. Because Perturb-seq captures a limited number of transcripts per cell, only the most highly expressed 5530 genes were analyzed. After filtering for differentially expressed genes, we found that the eIF3D cluster did not upregulate an entirely unique set of genes compared to other perturbations (Figure 5C). Instead, the clustering reflected subtle but coherent changes across several sets of genes. Compared to depletion of ESCRT subunits, genes in the eIF3D cluster promoted more modest levels of NF-κB activation. However, due to the relatively lower read-depth in Perturb-seq, none of the most highly upregulated immune-related transcripts (IL-8, IL-32, IFI6, CD83 and CD44) from bulk-RNA-seq of eIF3D knockdown cells were detected.

To test whether the transcriptional clustering defined by Perturb-seq would extend to lowly expressed genes, we performed bulk RNA-seq on cells expressing sgRNAs targeting eIF3D, eIF4G2, and eIF1A. Across all three genetic perturbations, we observed upregulation of 60/145 genes in the TNFα signaling via NF-κB annotation set (Figure 5D). Many of these genes were lowly expressed in wild-type K562 cells, with 37/60 expressed below 1 transcript per million (TPM). In addition, we observed coherent upregulation of 54/200 genes annotated as part of the interferon gamma response (Figure 5E), with only 10/54 genes overlapping with the TNFα signaling set. We confirmed that activation of NF-kB was not due to transcriptional repression of key NF-kB inhibitors (Supplementary Figure S5E). These data thus show that depletion of genes in the eIF3D cluster promotes a moderate innate immune response driven by NF-κB and interferon gamma.

Lastly, we investigated direction of causality between increased near-cognate start codon usage and NF-κB activation, as both occurred upon eIF3D depletion. Double knockdown of eIF3D and numerous key NF-κB signaling factors or TNFα factors yielded no changes to the CUG start codon reporter (Supplementary Figure S6A). Similarly, double knockdown with NFKBIA, the α-subunit of the inhibitory IKK complex (59), had no effect on the reporter phenotype. Overexpression of c-Jun, part of the AP-1 early response transcription factor complex (60), had no effect (Supplementary Figure S6B). We thus concluded that activation of NF-κB signaling upon eIF3D depletion was not responsible for increased near-cognate start codon usage.

## DISCUSSION

The fidelity of start codon selection plays a fundamental role in shaping the composition of the proteome. Transcripts contain up to hundreds of nucleotides upstream of the canonical start site that can potentially initiate translation from alternative reading frames. Initiation at upstream ORFs (uORFs) can play critical regulatory roles in the translation of their downstream partners, as in the integrated stress response (11,12). Furthermore, the peptide products from uORFs may sometimes exert functions independent from translational regulation (14).

We performed unbiased genome-wide CRISPRi screening to systematically identify the regulators of near-cognate start codon usage in human K562 cells. Our primary screen broadly recapitulated prior knowledge and identified known initiation factors eIF1, eIF1A, eIF5 and eIF5B. The majority of strong hits occurred in highly essential genes whose full knockouts are unviable, demonstrating the unique value of our CRISPRi approach in eliciting highly targeted knockdown phenotypes. Furthermore, we showed that depletion of all core eIF3 subunits and eIF3D in particular led to unusually robust near-cognate start codon usage.

While the exact mechanistic role of eIF3 and eIF3D in near-cognate start codon usage remains unclear, we ruled out alternative cap-binding by eIF3D/eIF4G2 and leucine tRNA initiation as potential mechanisms. Results from previous structural and biochemical studies provide avenues for future investigation. Biochemical characterization of eIF3D knockdown revealed that the loss of eIF3D does not compromise the integrity of the rest of the eIF3 complex (46,61). This unique property of eIF3D suggests that loss of eIF3D may influence scanning or decoding via conformational changes, whereas loss of other subunits leads to broader disruption of the eIF3 complex. In addition, our structure function data point to the N-terminal tail of eIF3D as being essential as opposed to its alternative cap-binding properties. These data also indicate that the effects of eIF4G2 on near-cognate usage are unlikely to be mediated by alternative cap-binding translation initiation (50). Recent cryo-electron microscopy structures have shown that the N-terminal tail of eIF3D physically interacts with eIF3C and eIF3E, connecting eIF3D to the rest of the eIF3 complex (57,58) suggesting that eIF3D depletion may allosterically affect the conformation of eIF3C, even if eIF3D depletion does not alter the overall assembly of eIF3. The altered conformation of eIF3C could then in turn tune the stringency of the decoding site during scanning via interactions with eIF1/1A and eIF5 (36,37). Establishing the exact structural effects of eIF3D depletion will require structural characterizations of the scanning complex in the absence of eIF3D or biochemical reconstitution of the initiation machinery with depleted levels of eIF3D.

Lastly, the downstream consequences of eIF3D knockdown extended beyond leaky translation initiation and included activation of NF-kB and cessation of growth. Depletion of other eIF3 subunits, eIF1A, or eIF4G2, induced a similar transcriptional response, suggesting that the activation of innate immunity could potentially be induced by altered start codon usage as opposed to depletion of eIF3 complexes alone. As many viral pathogens disrupt cellular translation, we speculate that the production of non-canonical ORFs could act as an intracellular signal that activates an antiviral response. It remains to be seen whether the inducer for such a response would involve *cis*-regulatory control of a master regulator by uORFs or whether increased production of specific uORF peptide products activate innate immune pathways.

## DATA AVAILABILITY

Raw sequencing data has been submitted to the NCBI SRA under accession numbers SRR19744356-SRR19744369. The corresponding BioSample accession numbers are SAMN29198687-SAMN29198700. Processed RNA-seq data are provided in the supplementary materials.

## Supplementary Material

gkad329_Supplemental_Files
