# Supplementary material for: Translational fidelity screens in mammalian cells reveal eIF3 and eIF4G2 as regulators of start codon selectivity
Source: Nucleic Acids Res. 2023 May 5;51(12):6355–69. doi: 10.1093/nar/gkad329 (PMC10325891; doi:10.1093/nar/gkad329)
Supplement: gkad329_Supplemental_Files [file gkad329_supplemental_files.zip › Supplemental Information_032723.docx]

**Supplementary Figures:**

**
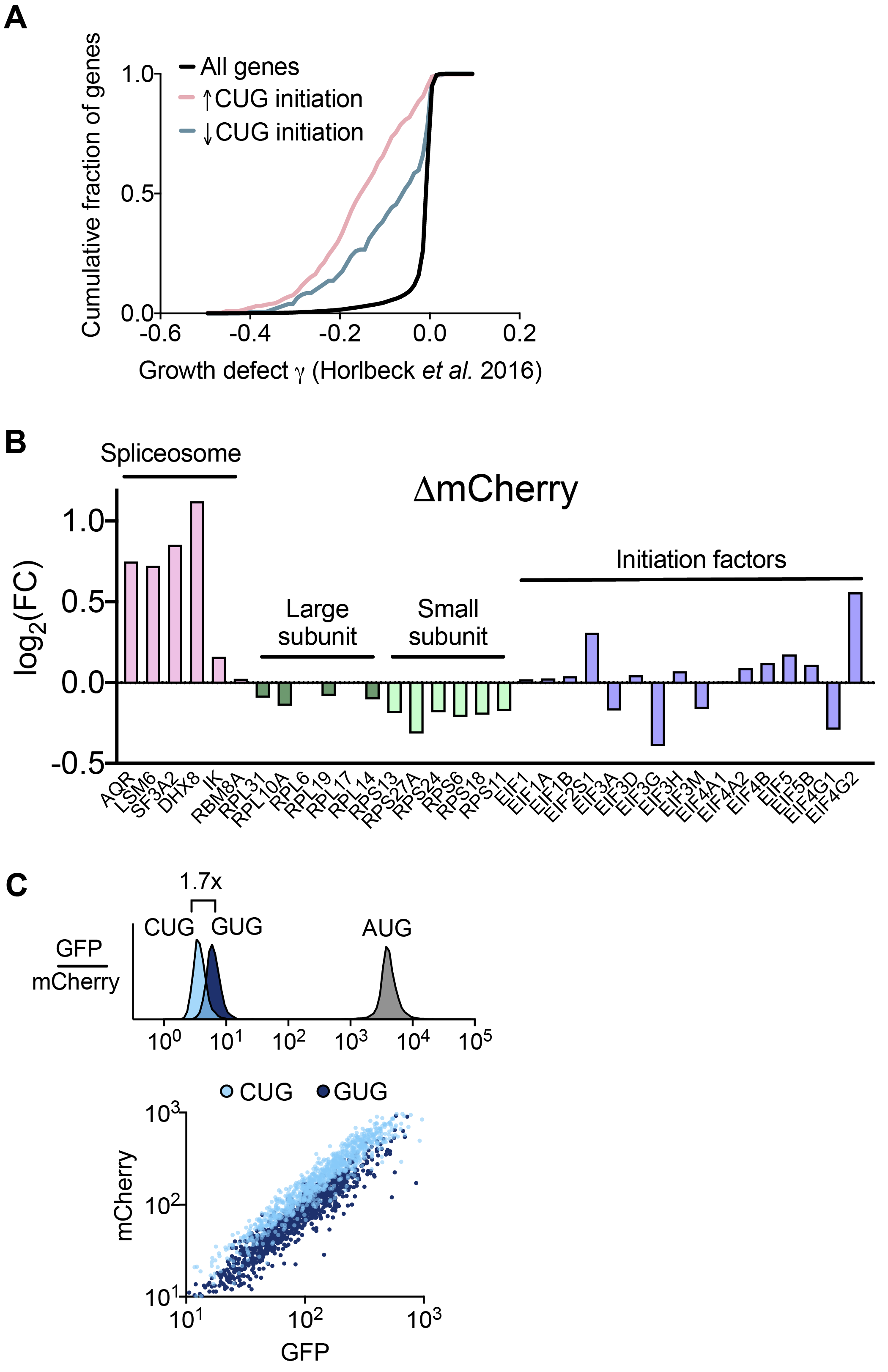
**

**Supplementary Figure S1**

(**A**) Cumulative distribution function of growth defect in K562 cells measured in (42) for all genes, genes that are enriched in the high GFP/mCherry fraction of the CUG near-cognate reporter, and genes that are enriched in the low GFP/mCherry fraction. (**B**) Change in IRES-driven mCherry expression upon sgRNA knockdown for major functional categories of sgRNAs in the 96 sgRNA validation screen. (**C**) GFP and mCherry fluorescence for polyclonal K562 cell lines with a CUG start codon reporter or GUG start codon reporter.

**
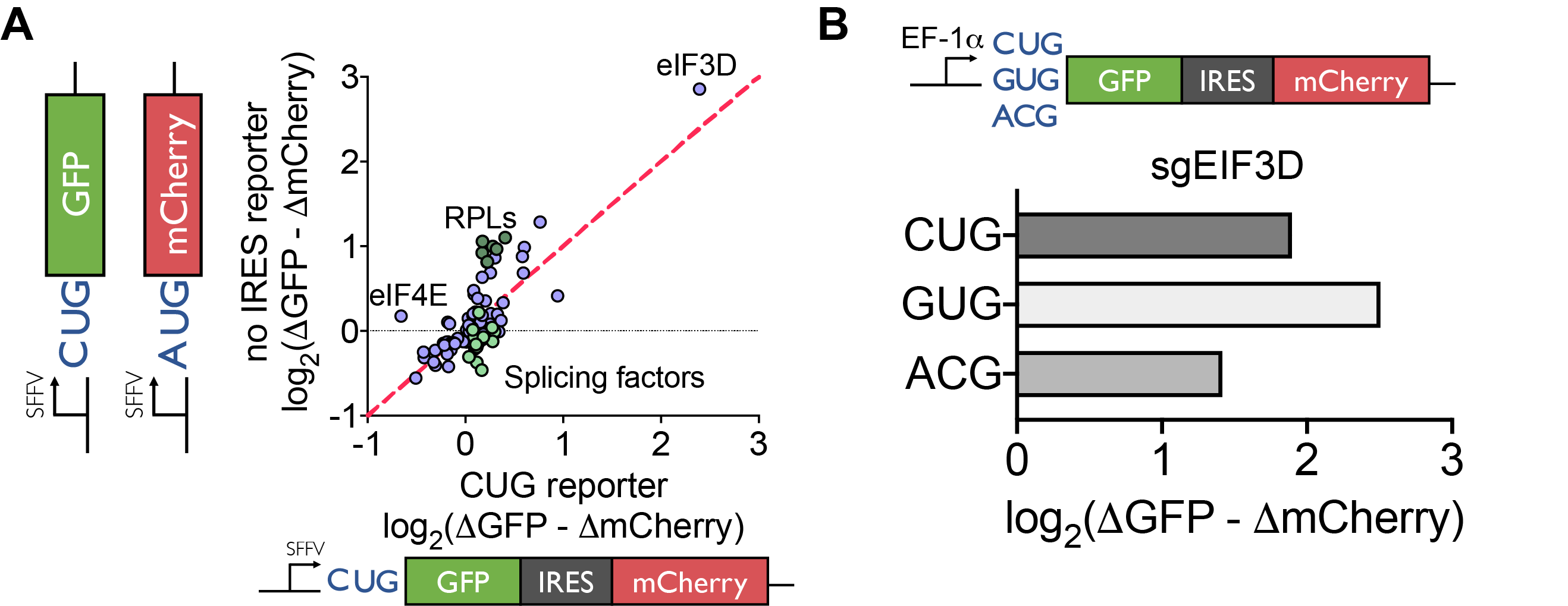
**

**Supplementary Figure S2**

(**A**) Comparison of individual sgRNA depletion phenotypes in cells expressing a CUG translation reporter vs. a no-IRES translation reporter with separate GFP and mCherry integrations. (**B**) eIF3D depletion phenotypes in near-cognate translation reporter variants driven by an EF-1α promoter, which contains a 5´terminal oligopyrimidine tracts (5´TOP) that potentially contributes to its eIF3D phenotype.

**
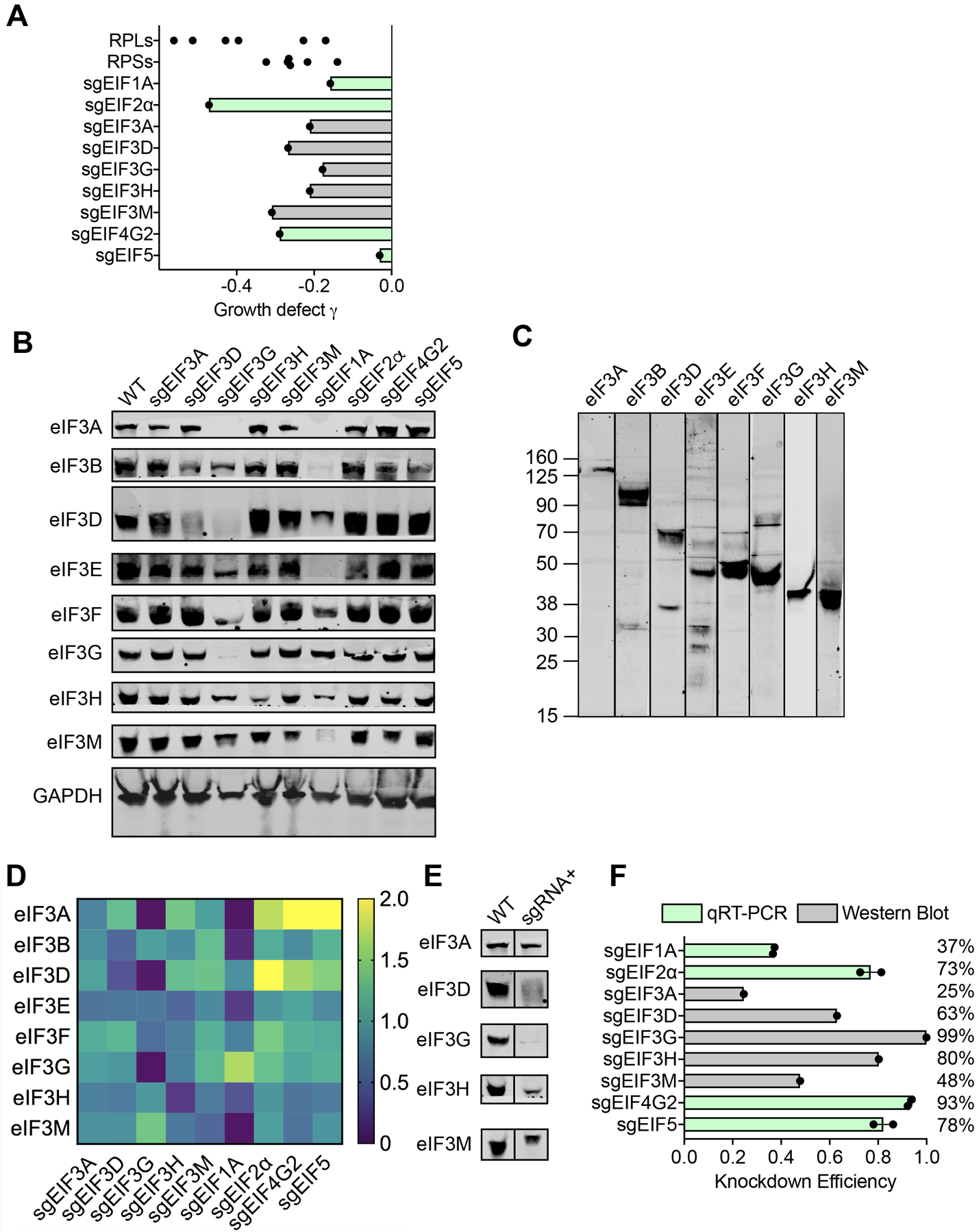
**

**Supplementary Figure S3**

(**A**) Growth defects for sgRNAs targeting major initiation factors compared to sgRNAs targeting ribosomal subunits, as measured by genome-wide growth screens in K562 cells (42). sgRNAs for initiation factors are the same as those used in western blot and qRT-PCR experiments. sgRNAs for ribosomal subunits are the full set of sgRNAs that were tested in the 96 sgRNA validation round (Supplementary Figure S2A). (**B**) Western blot on whole cell lysate for eIF3 complex subunits after 5 days of sgRNA mediated depletion. (**C**) Full-length blots for wild-type K562 cells for each eIF3 subunit antibody used in this study. (**D**) Quantification of western blot in (A) after normalization to GAPDH loading control for each lane. (**E**) Cropped western blot from (A) showing only bands from wild-type cells and the corresponding sgRNA target. (**F**) Combined qRT-PCR and western blot results quantifying the degree of knockdown efficiency for sgRNAs targeting major initiation factors.


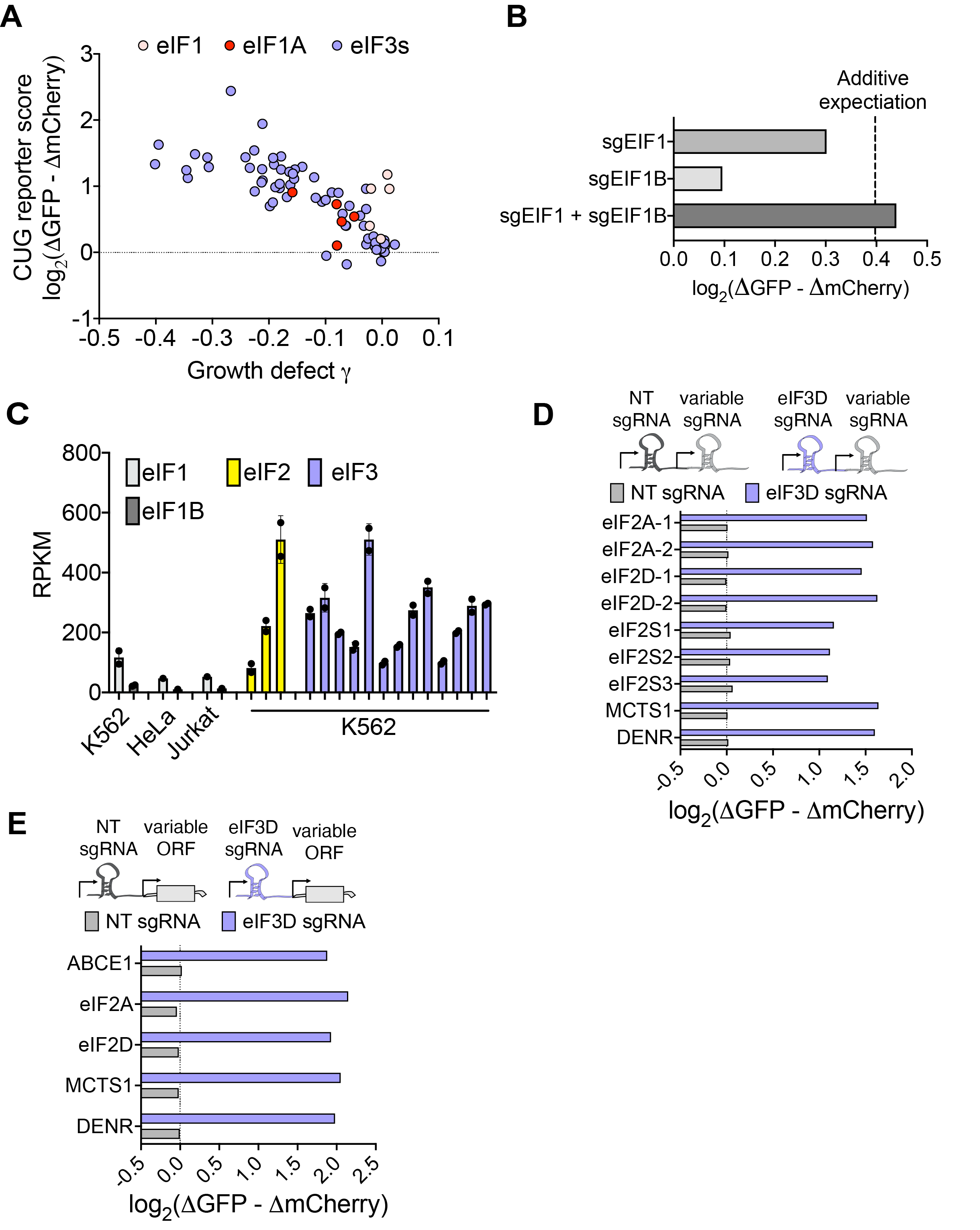


**Supplementary Figure S4**

(**A**) Scatter plot of CUG reporter score (log_2_(ΔGFP - ΔmCherry)) vs. growth defect γ (log_2_ sgRNA depletion per doubling) for sgRNAs targeting eIF1 and eIF1A. (**B**) The effect of double sgRNA knockdown of eIF1 and eIF1B in K562 cells on CUG reporter expression compared to individual sgRNA knockdowns. (**C**) Baseline mRNA expression levels (RPKM) of eIF1, eIF1B, eIF2, and eIF3 complex subunits. (**D**) CUG reporter expression with eIF3D knockdown + an sgRNA targeting an alternative initiation factors. eIF2A-1 and eIF2A-2 denote two separate sgRNAs targeting eIF2A, see table S1. (**E**) CUG reporter expression with eIF3D knockdown + overexpression of an exogenous ORF.

**
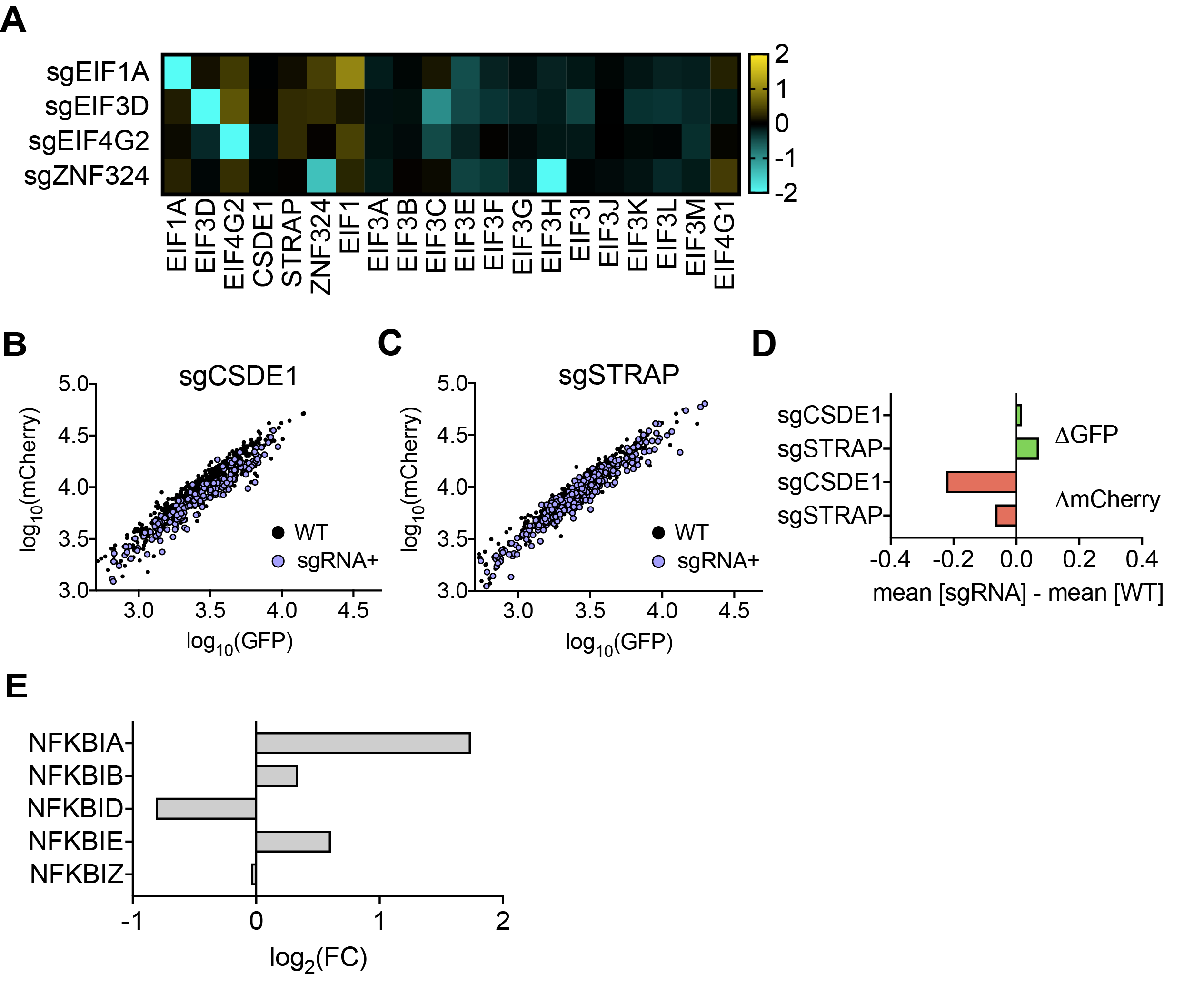
**

**Supplementary Figure S5**

(**A**) Bulk RNA-seq expression changes for K562 cells expressing sgRNAs targeting eIF1A, eIF3D, eIF4G2, and ZNF324. (**B**) GFP and mCherry expression levels in wild-type cells compared to cells expressing an sgRNA targeting CSDE1. (**C**) GFP and mCherry expression for cells with an sgRNA targeting STRAP. (**D**) Quantification of changes in mean GFP expression and mean mCherry expression for cells expressing CSDE1 or STRAP sgRNAs. (**E**) Bulk RNA-seq expression changes for K562 cells expressing eIF3D sgRNA for key NF-κB inhibitors.

**
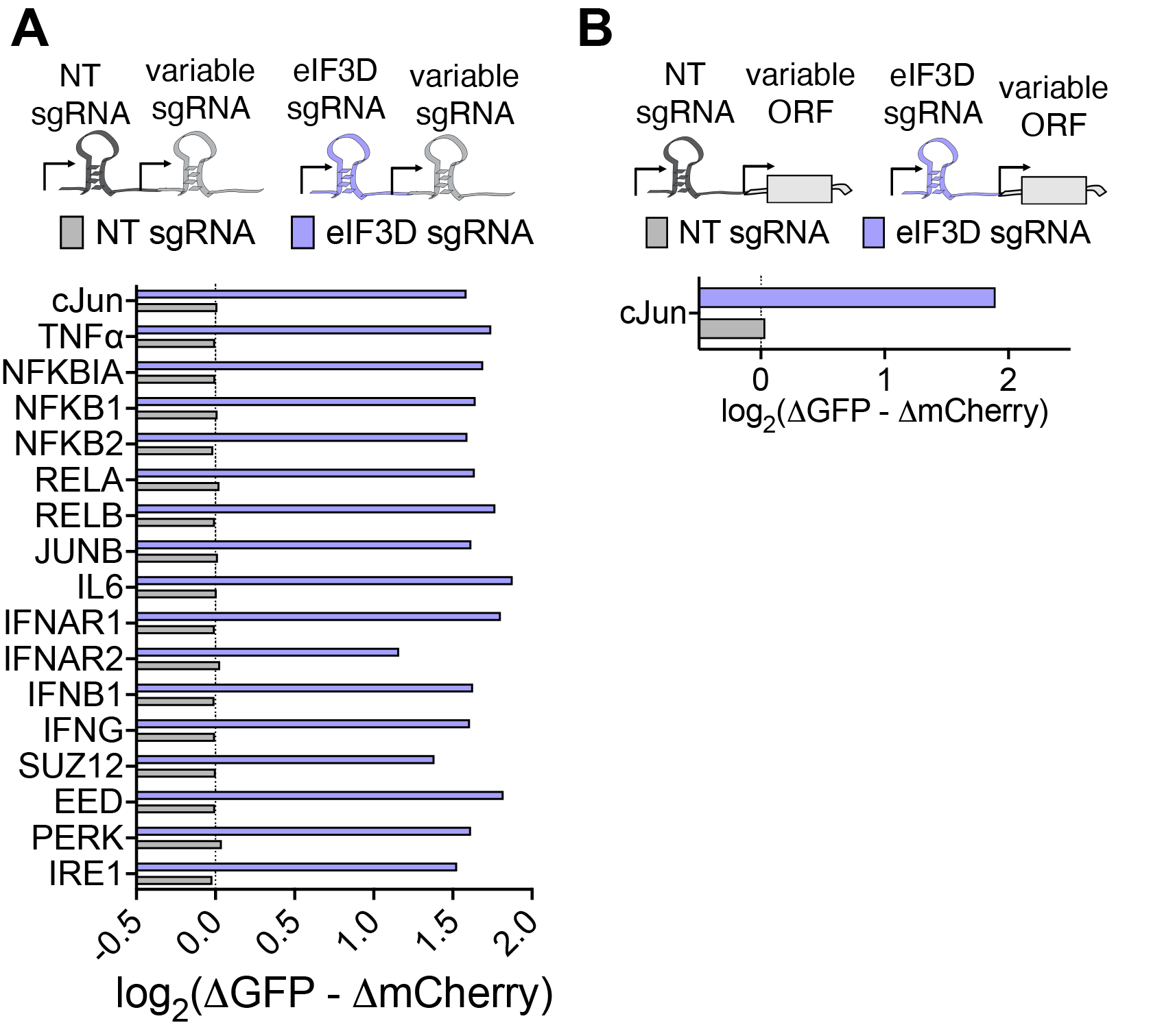
**

**Supplementary Figure S6**

(**A**) Measurements of CUG reporter expression upon double sgRNA knockdowns with an eIF3D sgRNA and an sgRNA targeting an NF-κB related gene. (**B**) CUG reporter expression with eIF3D knockdown + overexpression of an exogenous c-Jun ORF.

**Supplemental Table Legends**

**Table S1:** Primers used in this study.

**Table S2:** Antibodies used for western blots in Supplementary Figure S3.

**Table S3:** sgRNA and gene level enrichments in primary CUG reporter screen.

**Table S4:** List of 96 individual sgRNAs used for validation screening in Figure 2.

**Table S5:** Log_2_ fold-changes for CUG reporter with double sgRNA perturbations in Figure 3.

**Table S6:** List of 24 genetic interaction partner sgRNAs used in Figure 3.

**Table S7:** Bulk RNA-seq data for K562, Jurkat, and HeLa cells.
